# Effects of a Novel Starter Culture on Quality Improvement and Putrescine, Cadaverine, and Histamine Inhibition of Fermented Shrimp Paste

**DOI:** 10.3390/foods12152833

**Published:** 2023-07-26

**Authors:** Xinyu Li, Yang Zhang, Xinxiu Ma, Gongliang Zhang, Hongman Hou

**Affiliations:** 1 School of Food Science and Technology, Dalian Polytechnic University, No. 1 Qinggongyuan, Ganjingzi District, Dalian 116034, China; lixinyu990519@163.com (X.L.); zhangyang981223@163.com (Y.Z.); maxinxiu@hotmail.com (X.M.); zgl_mp@163.com (G.Z.); 2 Liaoning Key Lab for Aquatic Processing Quality and Safety, No. 1 Qinggongyuan, Ganjingzi District, Dalian 116034, China

**Keywords:** *Tetragenococcus muriaticus*, microbial community regulation, biogenic amine reduction, volatile compound, grasshopper sub shrimp paste

## Abstract

Fermented shrimp paste is a popular food in Asian countries. However, biogenic amines (BAs) are a typically associated hazard commonly found during the fermentation of shrimp paste and pose a food-safety danger. In this work, an autochthonic salt-tolerant *Tetragenococcus muriaticus* TS (*T. muriaticus* TS) strain was used as a starter culture for grasshopper sub shrimp paste fermentation. It was found that with the starter culture, putrescine, cadaverine, and histamine concentrations were significantly lower (*p* < 0.05) with a maximal reduction of 19.20%, 14.01%, and 28.62%, respectively. According to high-throughput sequencing data, *T. muriaticus* TS could change the interactions between species and reduce the abundance of bacterial genera positively associated with BAs, therefore inhibiting the BA accumulation during shrimp paste fermentation. Moreover, the volatile compounds during the fermentation process were also assessed by HS-SPME-GC-MS. With the starter added, the content of pyrazines increased, while the off-odor amines decreased. The odor of the shrimp paste was successfully improved. These results indicate that *T. muriaticus* TS can be used as an appropriate starter culture for improving the safety and quality of grasshopper sub shrimp paste.

## 1. Introduction

Shrimp paste is a traditional fermented aquatic food that is popular as a seasoning or side dish in several Southeast Asian and Chinese coastal areas due to its unique flavor and excellent nutritional value [1,2]. Among the various fermented shrimp pastes, grasshopper sub shrimp paste is special due to the particularity of its raw materials. Grasshopper sub shrimps, the most important ingredient, are krill-like shrimps with lengths of 8–10 mm that grow around the confluence of fresh and seawater. Therefore, grasshopper sub shrimp paste is a local specialty of regions around the Bohai Sea in China. Compared with pastes made of other shrimps, grasshopper sub shrimp paste is rich in astaxanthin and calcium contents, and low in fat and cholesterol levels due to the tiny size of the grasshopper sub shrimp [3]. However, the shrimp paste is usually produced at a small scale with a long fermentation period, and the quality of the shrimp paste is easily affected by environmental factors including temperature, the experience of operators, and microbiota [4]. In addition, the abundant amino acids present in shrimp paste provide sufficient substrates to produce some undesirable substances, especially biogenic amines (BAs).

As a kind of low-molecular-weight compound often found in fermented foods, BAs are usually created by microorganisms decarboxylating free amino acids and reducing ketones and aldehydes [5]. The consumption of dietary items containing high concentrations of BAs has a toxic effect on the human body, with symptoms such as headache, heart palpitations, vomiting, diarrhea, and hypertensive crises [6]. Among the common BAs in fermented foods, histamine (HIS) and tyramine (TRY) are considered to be the most dangerous substances, and they can cause toxic symptoms such as “scombroid fish poisoning” and “cheese reaction”, respectively [7,8]. The guidance level stated by the FDA for histamine is 50 mg/kg, and the maximum established by the European Food Safety Authority (EFSA) in fresh fish is 200 mg/kg [6]. Moreover, carcinogenic nitrosamines can be produced when cadaverine (CAD) and putrescine (PUT) combine with nitrite, and the toxicity of HIS can also be increased by the two BAs [9,10]. Therefore, the control and reduction of BAs are crucial for shrimp paste production.

Microbes involved in fermentation are vital to the development of the quality of fermented foods. As fermented aquatic food, rather complex microbial communities are engaged during the fermentation of shrimp paste [11]. These microorganisms produce a variety of enzymes and essential nutrients during the fermentation process, imparting the shrimp paste with unique flavor and nutrient characteristics [12]. According to earlier studies, the bacterial communities and interactions between species contribute to the quality and BA content of fermented aquatic foods [13,14]. Bacteria have served as starter cultures in several fermented foods to repress the accumulation of BAs and improve the flavor of the products [15,16,17,18,19]. It has been reported that the *Tetragenococcus* genus consists of moderately halophilic lactic acid bacteria (LAB) and significantly contributes to amino-acid production in several salted fermented foods [20]. Additionally, some *Tetragenococcus* species have been successfully used as starter cultures to reduce the accumulation of BAs, especially HIS [21,22]. Therefore, more knowledge about the mechanism of BA reduction is essential for providing theoretical references for the manufacture of safe, high-quality grasshopper sub shrimp paste products.

In our previous study, *Tetragenococcus muriaticus* TS (*T. muriaticus* TS), which predominates in grasshopper sub shrimp paste and produces fewer BAs, was isolated and subjected to safety assessment [23]. The strain displayed weak resistance to 15 known antibiotics based on the *Enterococcus* breakpoint values and exhibited no hemolytic activity and biofilm formation. Moreover, it performed well in salt tolerance, acid formation, and protease and lipase activities. Moreover, the strain showed positive amine oxidase activity and was able to degrade histamine in in vitro tests, therefore was considered to be a potential starter culture for grasshopper sub shrimp paste fermentation. Although the strain could produce PUT, CAD, and TRY (about 50 mg/kg in total) when supplemented with precursors in MRS broth, it was found to repress BA accumulation in grasshopper sub shrimp paste. In this study, *T. muriaticus* TS was used as a starter culture for the fermentation of grasshopper sub shrimp paste to reduce BA accumulation and improve the quality of shrimp paste in a short fermentation time. The contents of putrescine, cadaverine, and histamine were measured since they carry high risks for food safety. Moreover, the impacts of *T. muriaticus* TS on microbial communities were studied to further explore how the starter could reduce BA content from the perspective of the microbial community. The findings could provide a theoretical basis for the quality and safety control of shrimp paste products.

## 2. Materials and Methods

### 2.1. Chemicals and Reagents

Grasshopper sub shrimp was purchased from a local market in Panjin City, Liaoning Province, China. *Tetragenococcus muriaticus* TS was provided by this laboratory. Dansyl chloride was purchased from Dalian Meilun Bio-Technology Co., Ltd. (Dalian, China). Acetonitrile (chromatographic pure) was purchased from Sigma (Shanghai, China). All biogenic amine standards and derivatizing agents (dansyl chloride) were purchased from Sigma (St. Louis, MO, USA).

### 2.2. Preparation of Starter Culture

First, *Tetragenococcus muriaticus* TS (*T. muriaticus* TS) was cultured in a De Man Rogosa Sharpe (MRS) broth medium at 30 °C for 48 h. Then, the culture was centrifuged at 8000× *g* and 4 °C for 5 min to obtain bacterial cells (Centrifuge: Tomy MX-307, Tokyo, Japan). The cell pellets were then washed twice with sterile saline solution and resuspended in 0.05 mol/L phosphate buffer (pH 7.0). Subsequently, the suspension was adjusted to OD_600_ = 1.2 as the starter culture (5%, *v*/*w*).

### 2.3. Preparation of Grasshopper Sub Shrimp Paste Samples

The grasshopper sub shrimps caught in April 2022 were mixed with 15% (*w*/*w*) of salt. A total of 40 mL starter cell suspension was placed in a sealed jar containing 800 g of shrimp paste, and the same amount of shrimp paste combined with an equal volume of sterilized water was used as a control sample [14].

The shrimp paste placed in sealed jars was fermented at 30 °C for 35 days and stirred every day. Samples were collected on days 1, 7, 14, 21, 28, and 35. Considering the uniformity and representativeness of the paste, a total of 40 g was collected from the upper, middle, and lower parts of three different fermenters. Collected samples were combined for viable bacteria count analysis immediately, and the remaining samples were stored at −80 °C for further analysis. 

### 2.4. Physicochemical and Microbial Analysis

Samples (10 g) were homogenized in sterilized water (90 mL). Then, the homogenates were subjected to the determination of pH value using a pH meter (FiveEasy Plus^TM^ FE28, Mettler Toledo, Shanghai, China). The total volatile base nitrogen (TVB-N) and amino-acid nitrogen (AAN) contents were determined according to Chinese Standard GB5009.228 (2016) and GB5009.235 (2016), respectively. For the TVB-N, samples (5 g) were mixed with distilled water (75 mL) and allowed to stand for 30 min at room temperature. Thereafter, magnesium oxide (1 g) was added to the distillation tube containing the treated samples and immediately attached to the still of the Kjeldahl analyzer. The same volume of distilled water served as a control group. After that, the distillate was titrated to the endpoint with 0.1 mol/L hydrochloric acid solution. For the AAN, the mixed sample was quickly ground in a mortar, and then 5 g of the treated sample was diluted with distilled water to 100 mL, and the mixture was filtered. A total of 10 mL of filtrate mixed with 60 mL of distilled water was titrated to pH 8.2 with a sodium hydroxide standard solution (0.05 mol/L). Then, 10 mL of formaldehyde solution was added and the mixture was titrated to pH 9.2 with the sodium hydroxide standard solution.

Total aerobic mesophilic bacteria count and Lactic acid bacteria (LAB) plate count were conducted according to the method reported in previous research [16]. Briefly, 5 g samples were mixed with 45 mL sterilized saline in aseptic sampling bags and then homogenized by a stomacher (Stomacher 400 Circulator, Seward, Shanghai, China) for 2 min. Thereafter, decimal dilution was carried out and the suitable diluted solutions (200 µL) were added into the plate count agar (PCA) medium for the total aerobic mesophilic bacterial count, and MRS agar plates for lactic acid bacteria (LAB) count, and they were incubated in an aerobic environment at 37 °C for 48 h and 30 °C for 48 h, respectively. All experiments were repeated in triplicate.

### 2.5. Biogenic Amine Measurement by HPLC

The previously reported method was used to determine the level of putrescine, cadaverine, and histamine in the grasshopper sub shrimp paste samples [13]. In brief, 5 g of shrimp paste was mixed with 20 mL 10% trichloroacetic acid (TCA), and the mixture was homogenized by vortex mixing and stored at 4 °C to react for 2 h. Subsequently, the mixture was centrifuged at 3000× *g* and 4 °C for 10 min. Then, 200 µL of sodium hydroxide (2 M) and 300 µL of saturated sodium bicarbonate were added into 1 mL of the supernatant, followed by the addition of 2 mL of dansyl chloride solution (10 mg/mL), and the mixture was incubated at 45 °C for 40 min. A saturated sodium bicarbonate solution was obtained by adding a sufficient amount of sodium bicarbonate solid to distilled water until it could no longer be dissolved. After that, 125 µL of ammonia was added and the mixture was incubated at room temperature for 30 min to remove the residual dansyl chloride. The volume of the mixture was then adjusted to 5 mL with acetonitrile. Finally, the mixture was centrifuged at 3000× *g* for 5 min, and the supernatant was filtered twice through a 0.22 µm organic phase ultrafiltration membrane and stored at −80 °C for further HPLC analysis.

BAs were quantified using an Agilent 1260 HPLC unit (Agilent Technologies Inc., Santa Clara, CA, USA) consisting of an Agilent Zorbax SB-C 18 column (4.6 × 150 mm) with a quaternary pump and a diode array detector. A total of 20 µL of the sample was injected into the column at a flow rate of 1.0 mL/min. A binary solution consisting of ultrapure water (solvent A) and acetonitrile (solvent B) was used to elute the column using the following optimized gradient: 0–10 min, 55% B; 10–15 min, 55–65% B; 15–20 min, 65–80% B; 20–25 min, 80% B; 25–30 min, 80–90% B; 30–33 min, 90% B; 33–35 min, 90–55% B. The column temperature was set at 30 °C and the eluent was monitored by absorbance at 254 nm. LOD, RSD, and R^2^ are shown in Table 1. 

The quantification was accomplished by the external standard method, and the BA content was represented as mg/kg. All experiments were repeated in triplicate.

### 2.6. Microbial Community Analysis by Illumina High-Throughput Sequencing

Microbial community genomic DNA of grasshopper sub shrimp paste samples was extracted using the E.Z.N.A. soil DNA kit (Omega Bio-tek, Norcross, GA, USA) according to the instructions. The V3-V4 regions of bacterial 16S rRNA genes were amplified using the primer pair (338F and 806R) according to a previous method [3]. After PCR and purification, a DNA library was constructed and sequenced on the Miseq Illumina platform at Majorbio Bio-Pharm Technology Co., Ltd. (Shanghai, China). The raw sequences were spliced, and quality controlled by Flash software (version 1.2.11) and Fastp (version 0.19.6), and then OTU clustering (similarity 97%) was performed through Uparse (version 11) software. Species taxonomic annotation was conducted by an RDP classifier (version 2.13) based on the SILVA rRNA database (version 138). All samples were run with three repetitions.

### 2.7. Identification of Volatile Compounds by HS-SPME-GC-MS

Solid-phase microextraction (SPME; Supelco, Bellefonte, PA, USA) combined with 7890A-5975C chromatography–mass spectrometry (GC–MS; Agilent, Santa Clara, CA, USA) was used to determine the volatile compounds of samples collected at Day 35. After equilibration of 9 g samples in a 15 mL extraction flask at 70 °C for 15 min, 50/30 µm PDMS/CAR/DVB fiber (Supelco) was inserted into the flask (1 cm from the sample) for extraction of the volatiles at 70 °C for 45 min. Finally, the SPME device was inserted into the injection port of the GC, and desorption was carried out by exposing the fiber in splitless mode to 250 °C for 3 min. GC conditions were as follows: HP-5MS capillary column (30 m × 250 µm × 250 µm; Agilent); column oven temperature: 40 °C for 3 min, 3 °C/min to 120 °C, and finally increased at a rate of 5 °C/min to 240 °C, kept for 5 min; carrier gas: He; flow rate: 1.0 mL/min. MS conditions were listed as follows: electron ionization (EI) mode, voltage 70 eV, ion source temperature 230 °C, mass scanning range *m*/*z* 30–550. The volatile compounds (VCs) were identified by referring to their mass spectra in the NIST 14.0 database and by comparison with their retention index (RI). The relative percentages of each VC were calculated by dividing the peak area of the VC by the sum of the peak areas of all VCs.

### 2.8. Statistical Analysis

IBM SPSS Statistics 19 software was used for data analysis. The results were subjected to a one-way ANOVA analysis, with *p* < 0.05 considered to be statistically significant. Data were expressed as mean ± standard deviation. A part of the data was analyzed on the online platform of Majorbio Cloud Platform (http://www.majorbio.com (accessed on 15 July 2022)). All experiments were repeated in triplicate.

### 2.9. Nucleotide Sequence Accession Numbers

The bacterial 16S rRNA gene sequences obtained have been made publicly available in the NCBI Short Read Archive under the accession number PRJNA934728.

## 3. Results

### 3.1. Analysis of Bacterial Community in Shrimp Paste Samples

**Figure 1:**
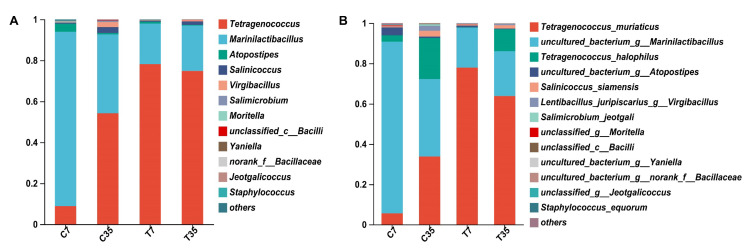
Comparative analysis of bacterial composition in control and test groups on genus (**A**) and species (**B**) level. Relative abundance of bacteria is presented as mean values, measured in triplicate. C: samples without starter culture; T: samples with starter culture. 7, 35: fermentation time (in days).

The bacterial composition in shrimp paste samples was determined by high-throughput sequencing and the results are shown in Figure 1. There were 11 genera identified in the control group and 7 genera in the inoculation group, respectively. The dominant genera in both groups were *Marinilactibacillus* and *Tetragenococcus*, with relative abundances of 85.13–38.47% and 8.83–54.16% in the control group and 19.85–22.17% and 78.13–74.82% in the test group, respectively (Figure 1A). After the starter culture inoculation, the relative abundance of *Marinilactibacillus* at 7 and 35 days decreased by 65.28% and 16.30%, respectively. In addition to the dominant genera, some genera with less abundance (<4%), such as *Virgibacillus*, *Atopostipes*, *Salinicoccus*, *Moritella*, *Jeotgalicoccus,* and *Staphylococcus*, were further reduced in relative abundance with the inoculation of starter culture (<2%).

Eight species were identified in both the control and test group. The predominant species in both groups were uncultured *Marinilactibacillus*, *T. muriaticus*, and *Tetragenococcus halophilus* (*T. halophilus*), with relative abundances of 85.13–38.47%, 5.63–33.78%, and 3.20–20.38% in the control group and 19.85–22.17%, 77.91–63.83%, and 0.22–10.99% in the test group, respectively (Figure 1B). After the starter culture inoculation, the relative abundance of *T. muriaticus* increased by 72.28% and 30.05% by Day 7 and Day 35, respectively. The relative abundance of uncultured *Marinilactibacillus* decreased by 65.28% and 16.30% by Day 7 and Day 35, respectively. The relative abundance of *T. halophilus* decreased by 2.98% and 9.39% by Day 7 and Day 35. In addition, some relatively low-abundance species, such as *Lentibacillus juripiscarius* and *Salinicoccus siamensis*, also decreased in relative abundance after the inoculation of the starter culture. The inoculation resulted in a significant difference in the abundance of the bacterial genera and species. 

**Figure 2:**
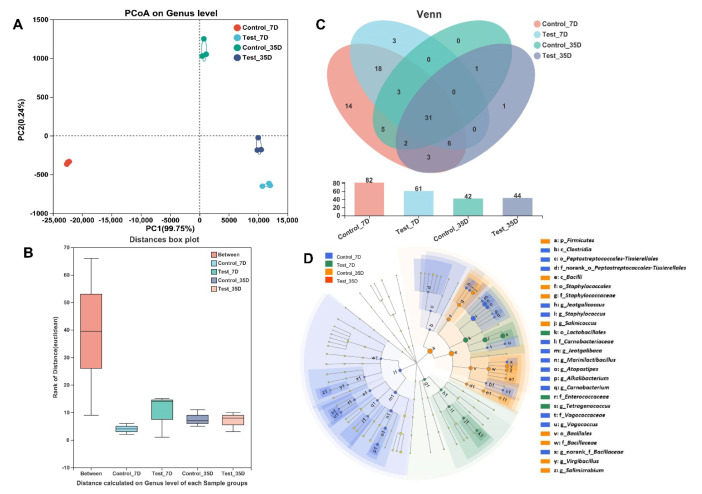
Comparative analysis of bacterial diversity at genus level between control and test groups. (**A**) PCoA of Euclidean. (**B**) Venn chart. (**C**) ANOSIM analysis. (**D**) LEfSe analysis of the microbial community in shrimp paste samples. PCoA: Different samples are represented as points with different colors and shapes. The closer the points are, the more similar the bacterial diversity of the samples. ANOSIM: The “Between” represents the difference between groups, and others represent the differences within each group. LEfSe: Bacteria significantly enriched in the corresponding group are represented by nodes with different colors, and the pale-yellow nodes represent bacteria with no significant difference in relative abundance among difference groups. Control and Test represent shrimp paste inoculated with/without starter, respectively. 7D and 35D: fermentation time (in days).

Principal coordinate analysis (PCoA), determined by the Bray–Curtis and Euclidean distance matrices, was conducted to explore the difference in bacterial diversity between the control and test groups, and the results are shown in Figure 2A. There was a large distance between the control and test group, while a small distance between the samples fermented for 7 and 35 days of the test group was found. The difference between groups was calculated to assess whether it was bigger than the difference within groups using analysis of similarities (ANOSIM), and it was discovered that the former was larger than the latter (Figure 2B). With operational taxonomic units (OTUs) identified at 97% sequence similarity, clustering was carried out to investigate the number of species in each group. A total of 87 OTUs were found at the genus level, as shown by the Venn diagram (Figure 2C). Totals of 83 and 68 OTUs were found in the control and test groups, representing 95.40% and 78.16% of the total OTUs, respectively. Linear discriminant analysis effect size (LEfSe) was conducted to find the bacteria with significant differences in relative abundance between control and test groups, and the result is shown in Figure 2D. The microbial biomarker in the test group was *Tetragenococcus*, while the microbial biomarkers found in the control group were *Marinilactibacillus*, *Lentibacillus*, and *Pseudoalteromonas*. The inoculation of *T. muriaticus* might have caused a significant change in the microbial diversity in the shrimp paste.

### 3.2. Effect of Starter Culture on the Interspecies Correlation

**Figure 3:**
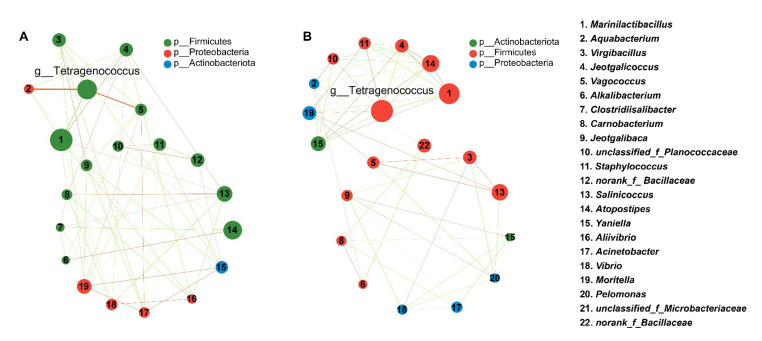
Comparative analysis of interspecies correlation of bacterial genus in control (**A**) and test (**B**) group. Correlations between bacteria are represented by lines between nodes. The red and green lines refer to positive and negative correlations, respectively.

The interactions between species were calculated to further investigate the impact of *T. muriaticus* on the microbial community in grasshopper sub shrimp paste. The interaction between different genera in shrimp paste samples changed significantly with the starter culture inoculation, and the number of genera correlated with *Tetragenococcus* increased from 5 to 8 (Figure 3A,B). By the 7th day of fermentation, the new genera positively correlated with *Tetragenococcus* in the test group were *Moritella*, *Staphylococcus,* and unclassified *Planococcaceae*, while the correlation of *Vagooccus* with *Tetragenococcus* disappeared after the starter inoculation. The new genus negatively correlated with *Tetragenococcus* was *Atopostipes*, and the correlation between *Virgibacillus* and *Tetragenococcus* disappeared. After the inoculation of *Tetragenococcus*, the negative correlation between *Tetragenococcus* and *Marinilactibacillus* was stronger. The inoculation of *T. muriaticus* changed the correlation among the dominant bacterial genera in shrimp paste.

### 3.3. Contribution of the Starter Culture to BA Reduction

**Figure 4:**
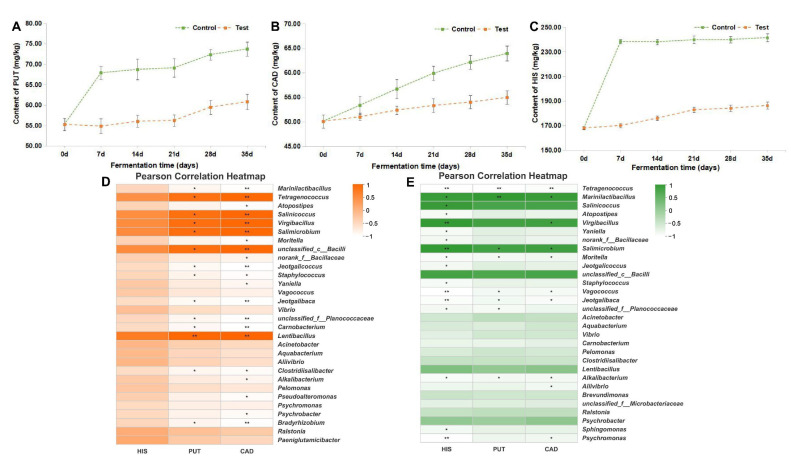
Content of putrescine (**A**), cadaverine (**B**), histamine (**C**) in shrimp paste samples and Pearson correlation analysis between bacteria genus and BAs in control (**D**) and test (**E**) groups. * 0.01 < *p* ≤ 0.05, ** 0.001 < *p* ≤ 0.01.

Changes in the content of putrescine (PUT), cadaverine (CAD), and histamine (HIS) were measured to explore the BA-degradation ability of *T. muriaticus*, and their contents are shown in Figure 4A–C, respectively. The contents of the three BAs increased continuously in both control and test groups during fermentation, and the inoculation of the starter culture was found to reduce their contents. The highest reduction of PUT and CAD were obtained as 19.20% and 14.01% by Day 7 and 35, respectively. The highest reduction of HIS occurred by Day 7 at 28.62%, and the content of HIS was reduced by 22.81% by Day 35. *T. muriaticus* was able to reduce the BA content when added to grasshopper sub shrimp paste. To further understand how *T. muriaticus* influenced the content of BAs from a microbial perspective, correlations between bacterial genera and the three BAs were conducted, and results are shown in Figure 4D,E. Interestingly, *Tetragenococcus* showed a significantly negative correlation with BAs in the test group while demonstrating a significant positive correlation with BAs in the control group.

### 3.4. Effects of Starter Culture on Physicochemical Properties and Microbial Counts of Shrimp Paste

**Figure 5:**
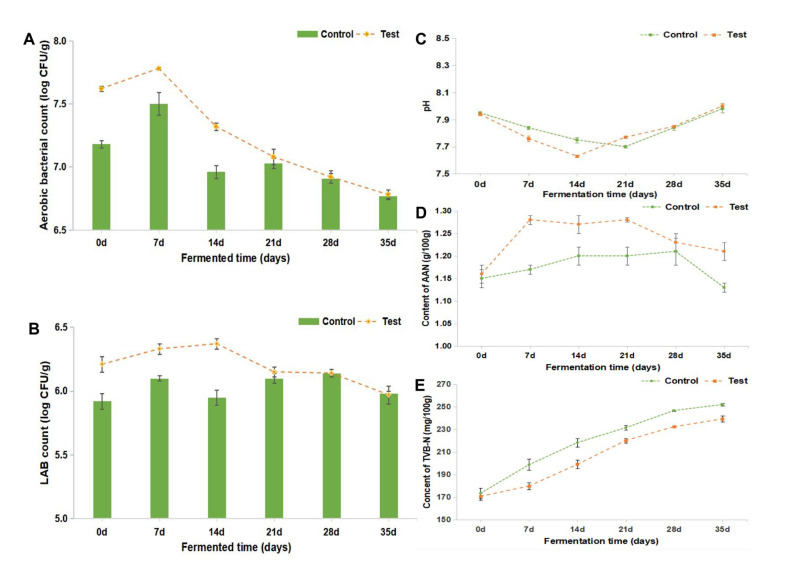
Microbiological and physicochemical characteristics of shrimp paste samples. (**A**) total aerobic bacterial count. (**B**) lactic acid bacteria count. (**C**) pH values. (**D**) Amino-acid nitrogen (AAN) content. (**E**) Total volatile base nitrogen (TVB-N) content.

The results of microbial counts are shown in Figure 5A,B. The total aerobic mesophilic bacterial counts on PCA agar plates grew gradually during the first 7 days and decreased gradually until the end of fermentation in both control and test groups. The highest bacterial counts in the control (7.47 log_10_ CFU/g) and test (7.75 log_10_ CFU/g) groups were both obtained on Day 7. The lactic acid bacteria (LAB) count of both control and test groups increased to the maximum value of 6.14 log_10_ CFU/g and 6.37 log_10_ CFU/g on Days 28 and 14, respectively, and then gradually decreased to 5.98 log_10_ CFU/g on Day 35. From Day 0 to Day 21, both the total aerobic mesophilic bacteria and LAB counts of the test group were consistently higher than those of the control group, with a difference of no more than 0.5 log_10_ CFU/g.

To investigate the impact of *T. muriaticus* on the physicochemical quality of shrimp paste, the pH values, total volatile base nitrogen (TVB-N), and amino-acid nitrogen (AAN) concentrations of shrimp paste throughout the fermentation were measured, and the results are presented in Figure 5C–E. The initial pH values of the control and test groups were both 7.9 (Figure 5C). The pH levels of shrimp paste first dropped to 7.6–7.7 and then quickly rose to 8.0. There was consistently more AAN present in the test group than in the control group throughout the whole fermentation. The content of AAN was measured since it was an indicator of the degree of fermentation of shrimp paste. The initial content of AAN in both groups was about 1.15 g/100 g (Figure 5D). The AAN content first increased to a relatively stable level and then decreased to 1.13 and 1.21 g/100 g in the control and test group, respectively. When compared to the control group, the AAN level in the test group was consistently higher. To assess the degree of amino-acid degradation, the content of TVB-N was examined throughout the fermentation process. The TVB-N concentrations of shrimp paste samples were about 170 mg/100 g before the fermentation began (Figure 5E). The TVB-N accumulated gradually in both batches throughout the fermentation processes and upon 35 days of fermentation, the values in the control and test group reached 252 and 240 mg/100 g, respectively. The TVB-N concentrations in both batches were lower than the limit (450 mg/100g) of the Chinese commercial standard for shrimp sauce (SB/T 10525-2009).

### 3.5. Impacts of Starter Culture on Volatile Compounds of Shrimp Paste

The variations in volatile components of shrimp paste samples collected on Day 35 were determined using the HS-SPME-GC-MS. Overall, a total of 25 volatile compounds (VCs), including 6 pyrazines, 1 hydrocarbon, 2 esters, 1 ether, 1 acid, 1 ketone, 2 alcohols, and 9 other compounds, were found in shrimp paste by GC–MS (Table 2). The most prominent VC type found in fermented shrimp paste was pyrazine, which comprised around 91.9% and 96% of the total VCs in the control and test groups, respectively. The esters and ethers were only identified in the control group, while acids, ketones, and alcohols were only found in the test group. It is worth noting that among the 9 other substances, amines were identified with an abundance of about 7% and 0.6% in the control and test group, respectively.

## 4. Discussion

Bacterial communities involved in the fermentation process are crucial for the quality of fermented shrimp paste. Previous studies have suggested that the dominant bacteria in fermented shrimp paste are *Pseudognonas*, *Staphylococcus,* and lactic acid bacteria such as *Tetragenococcus* and *Marinilactibacillus* [24,25]. In this study, *Tetragenococcus* and *Marinilactibacillus* with quite high abundance played a crucial role during shrimp paste fermentation, while *Staphylococcus* was low in abundance and contributed little to the fermentation process. It has been shown that *Tetragenococcus* is an important and predominant genus in grasshopper sub shrimp paste [13]. As a common lactic acid bacterium (LAB) in fermented foods, *Tetragenococcus* can grow well in environments with high salt concentrations, so they are crucial for the synthesis of several nutrients and flavor substances not only in shrimp paste but also in many fermented foods with high salt level [26,27]. *Marinilactibacillus* is a representative group of basophilic marine LAB and has been found in fermented herring and fermented rays [28,29]. A previous study has demonstrated that halophilic and alkaliphilic strains of *Marinilactibacillus* played a part in the fermentation of surface-ripened soft cheese and significantly influenced the quality of the product [30]. *Marinilactibacillus* might also play an important part in the quality formation of grasshopper sub shrimp paste, which was as rich in protein as cheese. The high-throughput sequencing data showed that the microbial composition in grasshopper sub shrimp paste has changed significantly with the inoculation of the starter culture. For the result of PCoA, the large distance between the control and test groups showed that the starter culture dramatically changed the microbial diversity in the shrimp paste, while the small distance between the samples fermented for 7 and 35 days of the test group indicated that the microbial community became more stable with the starter culture inoculation. Moreover, the results of ANOSIM and LEfSe also showed that the microbial diversity in the grasshopper sub shrimp paste changed significantly with the inoculation of the starter culture. For the species relevance network map, the more a node links to other nodes, the more important the node is [14]. The inoculation of *T. muriaticus* TS enhanced the impacts of *Tetragenococcus* during the fermentation process since there were more bacterial genera linked to the *Tetragenococcus* node in the test group than in the control group. The bacterial community changes might result in changes in the quality of the grasshopper sub shrimp paste during the fermentation process.

Excessive histamine (HIS) intake may result in food intoxication, which can cause symptoms including edemas, migraines, and low blood pressure. Although putrescine (PUT) and cadaverine (CAD) have far less toxicity, their interactions with amine oxidases may slow down the metabolism and increase the toxicity of histamine [31]. Controlling their content in food is therefore crucial for improving food safety. In our previous study, *T. muriaticus* TS showed histamine degradation of 12.60% in vitro. In this study, the histamine content decreased by 22.81% with the starter culture inoculation. A previous study has found that when the *Tetragenococcus halophilus* strain MJ4 was inoculated into fermented shrimp paste, the formation of cadaverine during the fermentation was repressed [20]. Our study demonstrated that *T. muriaticus* could enhance the food safety of shrimp paste by lowering the content of BA content produced during fermentation. LAB starters have been reported to inhibit the growth of bacteria with high BA-producing activities such as strains of enterococci, lactobacilli, streptococci, and lactococci [7]. Therefore, *T. muriaticus* might suppress the development of other LAB, therefore reducing the BA accumulation in the grasshopper sub shrimp paste.

The primary mechanisms for the synthesis of BAs in fermented foods are microbial amino-acid decarboxylation or amination and transamination of aldehydes and ketones. Therefore, correlations between bacterial genera and the three BAs were conducted. According to the results shown in the Pearson correlation heatmap, *T. muriaticus* could affect the correlation between the dominant bacterial genera and BAs (Figure 4D,E). *Tetragenococcus* was a dominating bacterium with a very high abundance in shrimp paste samples (Figure 1A), and the findings revealed that it positively correlated with BAs in the control group while negatively correlated with BAs in the test group. According to a previously published paper, *Tetragenococcus* was a dominant bacterium and positively correlated with BAs in fermented shrimp paste [23]. On the contrary, in our study, *Tetragenococcus* was negatively correlated with BAs in the test group. BA-producing capacity has been reported to be a strain-specific characteristic [5], and the *T. muriaticus* strain used in our work has been proven to suppress the accumulation of BAs in both vitro and grasshopper sub shrimp paste [23]. In the test group, *T. muriaticus* TS with BA-reducing ability grew rapidly and became the dominant strain, therefore the correlation between *Tetragenococcus* and BAs became inversely correlated. Moreover, although *Marinilactibacillus* was positively correlated with the three BAs in the test group, its abundance fell dramatically after the inoculation of the starter culture (Figure 1A).

The impacts of starter culture on the physicochemical quality of shrimp paste were also studied. Both the total aerobic mesophilic bacterial and LAB counts increased with the starter culture inoculation during the early fermentation phase, which might suggest that the starter culture could adapt efficiently to the fermentation environment. In addition, the increase in total aerobic mesophilic bacterial and LAB population was conducive to the formation of shrimp paste. The slight decrease in pH values during the early period of the fermentation process might be caused by the accumulation of lactic acid produced by LAB in the shrimp paste, consistent with the result of the LAB count (Figure 5B). The increase in pH values during the late fermentation stage might be due to the BAs or other basic substances produced by the excessive hydrolysis of proteins in shrimp paste [24]. In general, the inoculation of *T. muriaticus* TS had no significant effect on the pH values of the fermented shrimp paste. TVB-N is often employed as a physicochemical indicator to assess the freshness of aquatic items [32]. In general, a high TVB-N level means great destruction of amino acids. The data shows that *T. muriaticus* TS could significantly repress the accumulation of TVB-N and increase the content of AAN during the fermentation of grasshopper sub shrimp paste (*p* < 0.05). This could be brought on by the powerful capacity of *Tetragenococcus* for the enzymatic degradation of proteins during the fermentation of shrimp paste [24], and more proteins were broken down into nitrogen compounds existing in the form of amino acids rather than volatile base nitrogen compounds.

As an important characteristic, the odor changes in the shrimp paste were investigated by GC–MS. A strain of *T. muriaticus* has been used as a starter culture to influence the kind and abundance of key volatile components to enhance the volatile flavor of low-salt fish sauce [19]. In this work, the indigenous *T. muriaticus* TS strain was used for the grasshopper sub shrimp paste fermentation. According to the data of GC–MS, pyrazine, hydrocarbon, esters, ether, acid, ketone, and alcohol were identified as the volatile compounds in the grasshopper sub shrimp paste. These compounds have also been identified with different compositions and relative content in other fermented shrimp pastes. Shrimp species and fermentation processes may be responsible for the significant difference in the volatile compounds [25,33,34]. Pyrazines were the predominant volatile compound in shrimp paste samples and the starter culture increased the content of pyrazines in shrimp paste (Table 2). In this study, 3-ethyl-2,5-dimethyl-Pyrazine, trimethyl-Pyrazine, and 2,5-dimethyl-Pyrazine were the dominant pyrazine compounds with high content, consistent with previous studies [25,35]. Pyrazines are a crucial component of food flavor, and they have a significant impact on the sensory qualities of food. Due to their low taste threshold, they are crucial in the development of shrimp paste flavor and often provide a meaty, nutty, and roasted fragrance like roast potatoes [34]. Esters, ethers, acids, ketones, and alcohols were also identified in shrimp paste samples in this study (Table 2). However, they contributed less to the flavor of shrimp paste because they were relatively low in abundance (≤1.7%). It has been reported that some off-odor amine compounds were commonly produced by spoilage bacteria [36]. In this work, 2-Propanamine was identified in both control and test groups and Octodrine was found only in the control group, and the content of these two amines in grasshopper sub shrimp paste decreased by about 6.4% with the inoculation of the starter culture. These results demonstrated that *T. muriaticus* TS might improve the volatile flavor of grasshopper sub shrimp paste by enhancing the aroma brought on by pyrazines and reducing the unpleasant odor caused by amines.

## 5. Conclusions

Autochthonic salt-tolerant *Tetragenococcus muriaticus* TS (*T. muriaticus* TS) was successfully used as a starter culture to decrease BAs content. Contents of putrescine (PUT), cadaverine (CAD), and histamine (HIS) decreased significantly with the inoculation of *T. muriaticus* TS, reaching the maximal reduction at 19.20%, 14.01%, and 28.62%, respectively. The overall odor of the grasshopper sub shrimp paste was improved by the increase of pyrazines and the decrease of amines. Results of high-throughput sequencing demonstrated that the inoculation of *T. muriaticus* TS could interfere with the bacterial structure and interspecies correlation. Correlation analysis found that the inoculation of *T. muriaticus* TS could suppress the growth of bacteria that were positively correlated with BAs, thus reducing the BA accumulation in grasshopper sub shrimp paste. In summary, inoculation of starters such as *T. muriaticus* TS was an effective method to produce grasshopper sub shrimp paste with higher quality and lower food-safety risks caused by high levels of BAs.

## Figures and Tables

**Table 1:** Validation parameters of the HPLC method.

Biogenic Amines	LOD/(mg/kg)	RSD/%	Regression Equation	R^2^
PUT	0.80	2.36	y = 29.554x − 5.7576	0.9991
CAD	0.80	2.74	y = 30.636x − 32.384	0.9993
HIS	1.20	1.24	y = 26.431x − 26.907	0.9990

**Table 2:** Volatile compounds identified by HS-SPME-GC-MS in shrimp paste samples.

No.	RT (min)	Compound Name	Relative Percentage (%)	
			Control	Test
Pyrazines				
A1	15.09	3-Ethyl-2,5-dimethyl-pyrazine	55.40	56.31
A2	11.41	Trimethyl-pyrazine	17.08	18.73
A3	7.11	2,5-Dimethyl-pyrazine	16.50	17.45
A4	10.45	2,6-Dimethyl-pyrazine	0.27	0.20
A5	19.48	3,5-Diethyl-2-methyl-pyrazine	2.64	ND
A6	19.31	2,3,5-Trimethyl-6-ethylpyrazine	ND	3.27
Hydrocarbons				
B1	1.89	Oxirane, 2-butyl-3-methyl-, cis-	0.22	ND
B2	37.35	Hexa-t-butylthiatrisiletane	ND	0.03
B3	38.29	Silane, [[4-[1,2-bis[(trimethylsilyl)oxy] ethyl]-1,2-phenylene] bis(oxy)] bis [trimethyl-]	ND	0.04
Esters				
C1	5.08	Arsenous acid, tris(trimethylsilyl) ester	0.49	ND
C2	32.58	Benzenesulfonic acid, 4-methyl-, bicyclo [3.2.1] oct-6-yl ester	0.05	ND
Ethers				
D1	38.29	18-Methyl-nonadecane-1,2-dio, trimethylsilyl ether	0.06	ND
Acids				
E1	1.59	12-Methylaminolauric acid	ND	1.36
Ketones				
F1	25.76	Thebacon	ND	0.07
Alcohols				
G1	1.40	Cyclobutanol	ND	1.62
G2	51.51	trans-Farnesol	ND	0.18
Other compounds				
H1	1.42	2-Propanamine	3.82	0.59
H2	1.61	Octodrine	3.20	ND
H3	3.78	1,2,4-trimethyl-Piperazine	0.17	ND
H4	49.20	4H-1-Benzopyran-2-carbonyl chloride, 4-oxo-	0.04	ND
H5	25.76	2-Chloro-4-(4-methoxyphenyl)-6-(4-nitrophenyl) pyrimidine	0.08	ND
H6	3.75	1,3,3-Trimethyl-diaziridine	ND	0.07
H7	29.84	1-Ethoxy-2-methyl-benzene	ND	0.06
H8	47.68	5-Fluoroorotyl-N, N-dimethylhydrazide	ND	0.01
H9	52.08	1H-Indole, 2-(1,1-dimethyl-2-propenyl)-6-(3-methyl-2-butenyl)-	ND	0.01

ND: Not detected. RT: Retention time in the capillary column. Control and Test represent shrimp paste fermented without/with starter culture for 35 days, respectively.

## Data Availability

The data presented in this study are available on request from the corresponding author. The data are not publicly available due to the need for the first author to the apply for a master’s degree.
